# Activity-based protein profiling of rice (*Oryza sativa* L.) bran serine hydrolases

**DOI:** 10.1038/s41598-020-72002-w

**Published:** 2020-09-16

**Authors:** Achintya Kumar Dolui, Arun Kumar Vijayakumar, Ram Rajasekharan, Panneerselvam Vijayaraj

**Affiliations:** 1 Lipid and Nutrition Laboratory, Department of Lipid Science, CSIR-Central Food Technological Research Institute, Mysuru, Karnataka 570020 India; 2 Academy of Scientific and Innovative Research, Ghaziabad, Uttar Pradesh 201002 India; 3 CSIR-Central Food Technological Research Institute, Resource Centre Lucknow, Lucknow, 226018 India; 4 School of Life Sciences, Central University of Tamil Nadu, Tamil Nadu, Neelakudi, Thiruvarur, 610 005 India

**Keywords:** Biotechnology, Plant sciences

## Abstract

Rice bran is an underutilized agricultural by-product with economic importance. The unique phytochemicals and fatty acid compositions of bran have been targeted for nutraceutical development. The endogenous lipases and hydrolases are responsible for the rapid deterioration of rice bran. Hence, we attempted to provide the first comprehensive profiling of active serine hydrolases (SHs) present in rice bran proteome by activity-based protein profiling (ABPP) strategy. The active site-directed fluorophosphonate probe (rhodamine and biotin-conjugated) was used for the detection and identification of active SHs. ABPP revealed 55 uncharacterized active-SHs and are representing five different known enzyme families. Based on motif and domain analyses, one of the uncharacterized and miss annotated SHs (Os12Ssp, storage protein) was selected for biochemical characterization by overexpressing in yeast. The purified recombinant protein authenticated the serine protease activity in time and protein-dependent studies. Os12Ssp exhibited the maximum activity at a pH between 7.0 and 8.0. The protease activity was inhibited by the covalent serine protease inhibitor, which suggests that the ABPP approach is indeed reliable than the sequence-based annotations. Collectively, the comprehensive knowledge generated from this study would be useful in expanding the current understanding of rice bran SHs and paves the way for better utilization/stabilization of rice bran.

## Introduction

Rice (*Oryza sativa*) is one of the major staple foods for almost half the world’s population, especially in Asia^1^. In rice seed, the integrated portion of endosperm and germ are the vital source of energy mainly in the form of starch. The rice bran (aleurone layer) of rice seed contains nutraceutical compounds like γ-oryzanol, phytosterols, tocopherols, and tocotrienols with 15 to 20% oil^2, 3^. Rice bran oil is considered as one of the healthiest oil due to its unique and desirable fatty acid composition and health-enhancing compounds. However, the presence of endogenous lipases and hydrolases are responsible for the rapid deterioration of rice bran. In *O. sativa,* several genes encoding serine hydrolases (lipases/protease) have been reported in the bran^4–9^. Bhardwaj et al.^6^ reported a thermostable rice bran lipase with phospholipase activity. So far, there are no reports or attempts made to provide comprehensive profiling of active SH enzymes present in rice bran. It could be due to the lack of a suitable analytical platform to screen, identify, and isolate the active enzymes present in native complex biological systems. Since high-quality genome assemblies are well established in rice for plant biology and agricultural research, *O. sativa* constitutes model systems for Monocotyledons^10^. However, a major challenge is to assign the function to the > 35,000 proteins encoded by the *O. sativa* genome^11^. Several genome-wide technologies (transcriptomes and proteomes) have provided remarkable information about *O. sativa* genomes, and insights into diverse biological processes^12–15^. But the functional and biochemical characteristic is a crucial link that is lacking between the proteome and the annotated metabolic processes. Since the enzyme activities depend on the various post-translational modifications, assigning enzyme activities from abundance-based transcriptomics or proteomics data could be misleading^16^. Moreover, the sequence-based functional annotation is not reliable in the case of more divergent proteins^17^. Hence, the functional annotation of proteins in an activity-dependent manner is crucial for understanding their roles in living systems. With the advent of Activity-based protein profiling (ABPP), the functional annotation is possible in the native biological systems^18, 19^. ABPP employs active site-directed chemical probes (conjugated with fluorescent or biotinylated tag) that covalently binds large numbers of mechanistically related enzymes^18, 20, 21^. In plants, Serine hydrolases (SHs) play a significant role in virtually all physiological processes. It consists of a wide range of enzymes that carry an activated serine residue in their catalytic site. SHs are typically hydrolytic enzymes such as proteases, lipases, esterases, including acyltransferases^22, 23^. In plants, SHs were reportedly involved in the regulation of stomatal density (e.g., SDD1), immune responses against various conditions (e.g., GDSL lipases, and proteases), detoxification processes (e.g., carboxylesterase CXE12) and production of secondary metabolism (e.g., acyltransferase SNG1)^24–26^. SHs particularly, proteases and lipases, have a crucial role during seed maturation as well as germination^27, 28^. Activation of SHs involves in various hydrolytic enzyme activities engaged in the energy mobilization to ensure a proper post-germinative growth to establish photosynthetically active seedling with healthy root and shoot system^29^. In rice, the maximum hydrolase activity was reported in bran^30^. Hence, we attempted to provide the first comprehensive profiling of active serine hydrolases present in *O. sativa* bran proteome by the ABPP approach. Further, the biochemical characterization of the 12S storage protein was demonstrated as a serine protease in this study.

## Results

### Detection of rice bran SHs by in-gel fluorescence scanning

**Figure 1:**
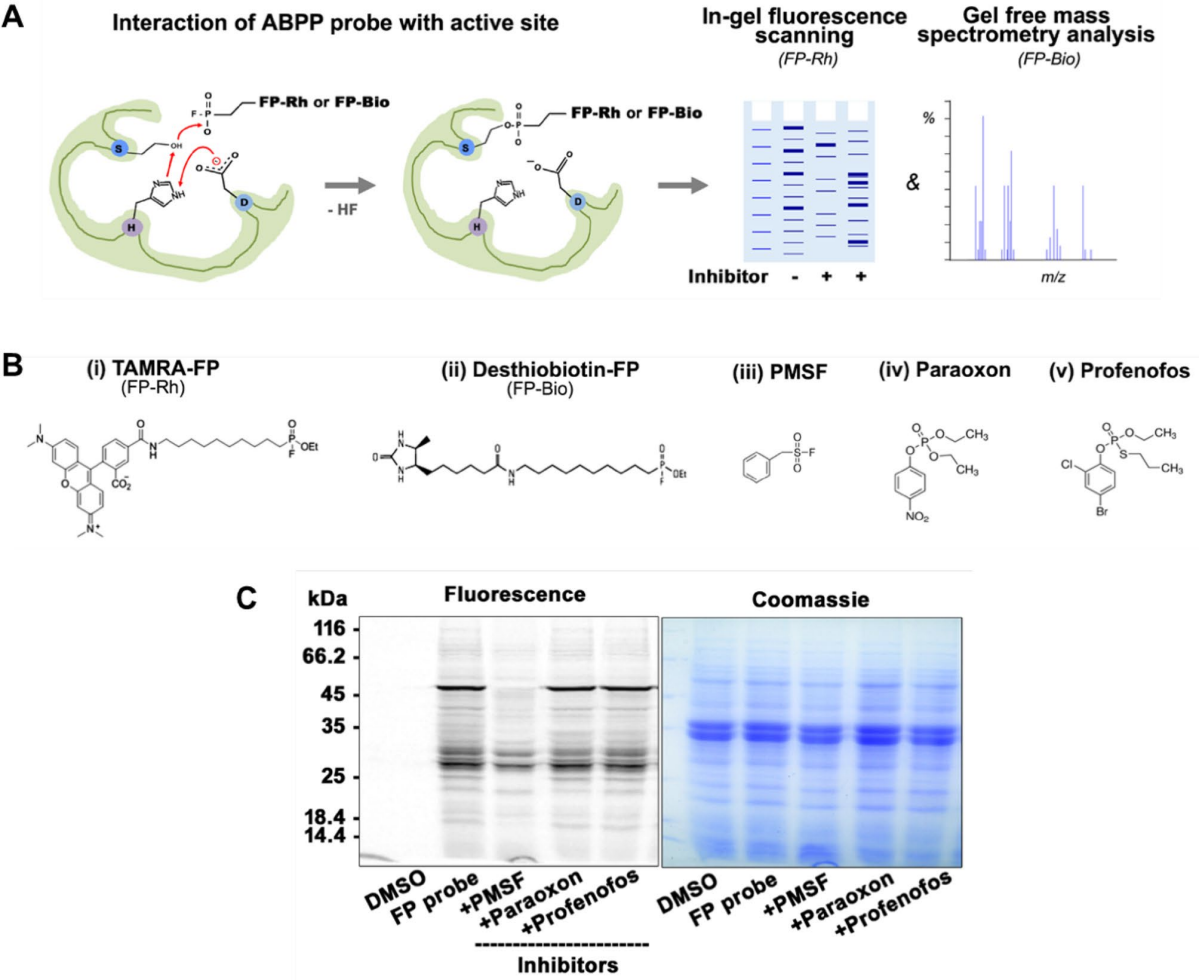
In-gel fluorescence labeling of rice bran serine hydrolases. (**A**) Schematic representation of activity-based protein profiling workflow. The ABPP probe (Fluorophosphonate, FP) interact and covalently binds to serine in the active site of the proteins. The interaction of the probe with proteins is detected by either in-gel fluorescence scanning (rhodamine as a fluorophore) or LC–MS analysis using biotinylated probes. (**B**) Structures of the FP-probes, and the reported serine hydrolase inhibitors used in the in vitro competitive ABPP assay. (i) Rhodamine conjugated fluorescent fluorophosphonate probes (TAMRA-FP), (ii) Desthiobiotin conjugated FP, (iii) PMSF, (iv) Paraoxon, and v) Profenofos. (**C**) In-gel fluorescence labeling of rice bran protein. Rice bran proteins were labeled by incubating a 2 μM FP-serine hydrolase probe for 1 h at 37 °C. After labeling, proteins were separated on a 12% SDS-PAGE, and fluorescent intensity was detected by fluorescence scanning. For in-vitro competitive ABPP profiling, RB proteins were pre-incubated with inhibitor or DMSO (no probe control) at 37 °C for 30 min, followed by labeling with FP-probe for 1 h. Inhibitors compete with FP-probes for enzyme active sites, leading to a loss of fluorescence intensity. The competition is read out by fluorescence scanning, followed by Coomassie brilliant blue staining to visualize the complete protein profile (right panel).

Activity-based proteomics is an analytical platform ideally suited for the global profiling of protein function^31^. In this approach, the functional annotation of uncharacterized proteins can be identified based on its activity-dependent. A typical ABPP workflow is shown in Fig. 1A. The detection and identification of rice bran SHs were performed by in-gel fluorescence scanning. The active site-targeted chemical probe interacts and covalently binds to the active site of SHs in the proteome. The probe labeled SHs are then detected by in-gel fluorescence scanning (FP-Rh; Fig. 1B-i). After labeling, proteins were separated on SDS-PAGE, followed by in-gel fluorescent scanning at 532 nm. The labeled proteins were detected in the soluble fraction with strong signals at 50 and 27 kDa (Fig. 1C, lane 2). Apart from these two major signals, a number of weaker signals were also observed. The labeling patterns showed the presence of active SHs at the various masses. To get more insights into the specificity and the selectivity of these SHs, we adopted an in vitro competitive ABPP with known serine hydrolase inhibitors such as PMSF (Fig. 1B-iii), Paraoxon (Fig. 1B-iv), and Profenofos (Fig. 1B-v). These inhibitors compete with the FP-Rh probe for active sites of SHs, and this competition was visualized by the loss of the fluorescence signal. DMSO was included as a no probe control. As expected, PMSF reduced the overall reduction of signals (Fig. 1C, lane 3) as compared to no inhibitor lane (Fig. 1C, lane 2). Interestingly, pre-incubation of protein lysate with PMSF completely abolished the signal at 50 kDa and ~ 50% at 27 kDa protein. These results indicate that these two proteins could be protease (Fig. 1C, lane 3). There was also a significant reduction in the labeling pattern of other polypeptides. Both esterase inhibitors (paraoxon and profenofos) treated samples showed a similar labeling pattern with a reduction in signal. However, the maximum decrease in labeling was observed with paraoxon treatment (Fig. 1C, lane 4). Interestingly, these esterase inhibitors had no effect on the labeling of both 50 and 27 kDa proteins, which further confirmed that these two proteins could be serine proteases. In vitro*,* ABPP assay indicated that there were many putative serine hydrolases active during the assay conditions, and these were sensitive to the tested inhibitors. However, the in-gel fluorescence ABPP does not provide a molecular identity to the probe-labeled target proteins^32^.

### Identification of probe targets by gel-based ABPP

**Figure 2:**
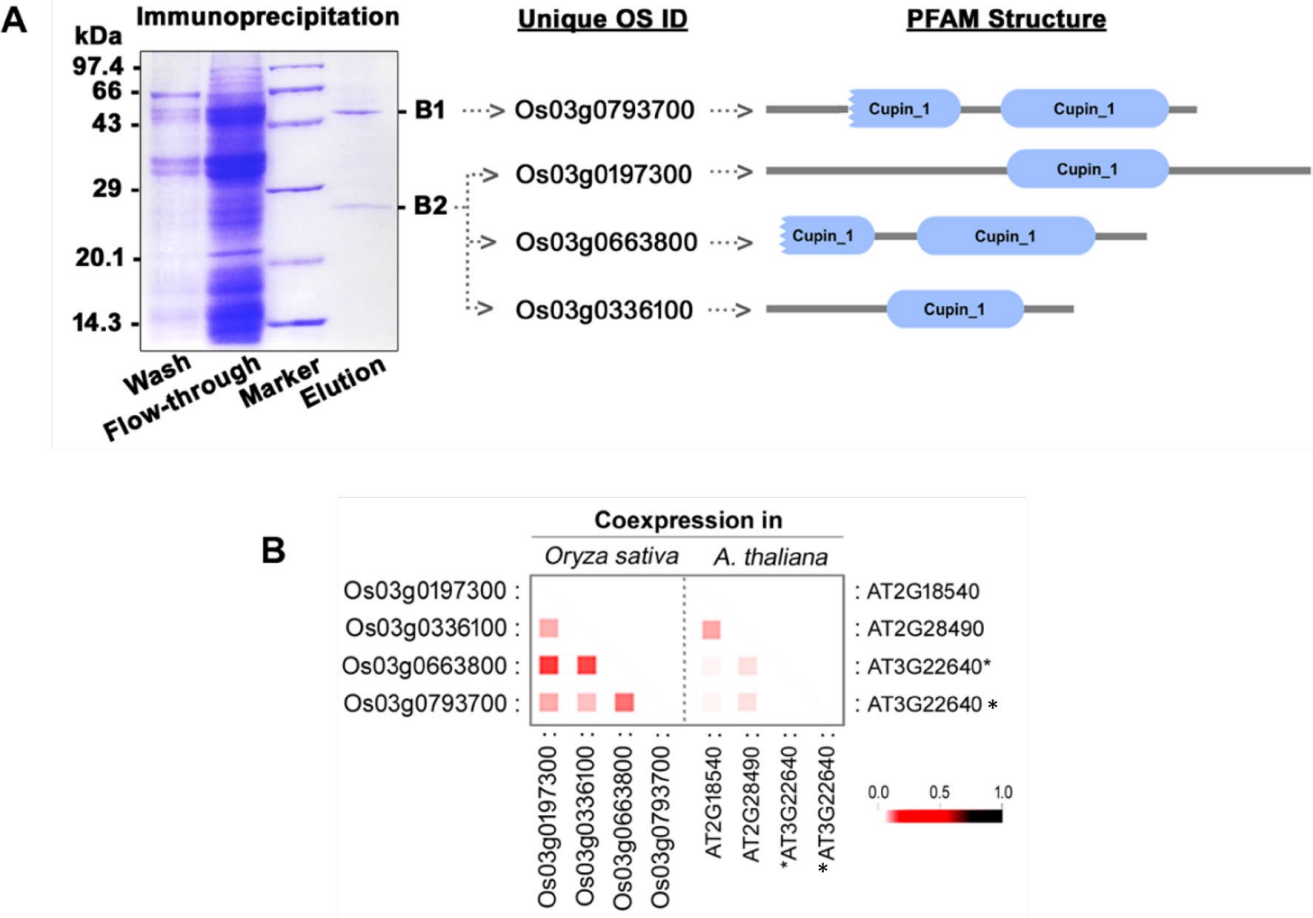
Identification of major rice bran serine hydrolases. (**A**) Enrichment of two major rice bran (RB) serine hydrolases by immuno-pulldown assay followed by LC–MS analysis. RB protein lysate was incubated with fluorophore-conjugated probes (TAMRA-FP) followed by the addition of an anti-TAMRA monoclonal antibody. The enzyme-antibody complex was captured by protein-A Agarose beads. The eluted fraction was separated on a12% SDS-PAGE, and the major bands (B1 and B2) were excised for in-gel trypsin digestion followed by Mass spectrometry analysis. The unique OS ID of the identified proteins is shown along with PFAM structures. (**B**) Coexpression analysis of the identified proteins using STRINGdb. The co-expression pattern of the B1 and B2 proteins was observed in *O. sativa* and its closest homolog in *Arabidopsis thaliana*. All the proteins showed coexpression. The intensity of the red square represents a higher association with each other. *AT3G22640 is a homolog of Os3g0663800 and Os3g0793700.

To get the identity of the labeled proteins, first, we performed the immuno-pull down experiment followed by classical in-gel trypsin digestion. In this approach, the RB proteins were incubated with the FP-Rh probe followed by incubation with anti-TAMRA (anti-fluorophor) monoclonal antibody^33^. The labeled protein-antibody complex was purified using affinity beads. The enriched proteins were separated on SDS-PAGE, which reveals the presence of two major proteins at 50 and 27 kDa regions (Fig. 2A). The protein profile was similar to the in-gel fluorescence labeling of SHs (Fig. 1C). The 50 kDa and 27 kDa bands were named B1 and B2, respectively. After in-gel trypsin digestion and mass spectrometry, the Mascot analysis revealed that B1 corresponds to NP_001051533.1 protein with a predicted molecular weight of 52 kDa. The corresponding gene encoding this protein is Os03g0793700 (unique OS ID). B1 protein is annotated as a cupin domain-containing protein. The competitive ABPP assay with PMSF also revealed that the labeling of Os03g0793700 was abolished (Fig. 1C, lane 3). The identified cupin domain containing SH may have functional property, specifically protease activity. The second predominant band B2 at 27 kDa corresponds to three cupin domain-containing proteins. It was identified as a cupin domain containing globulin like protein NP_001049271.1 (Unique OS ID, Os03g0197300) with a predicted molecular weight of 63 kDa. Moreover, the FP-Rh labeling of this protein was blocked by PMSF (Fig. 1C, lane 3). Based on the observed results, we predicted both B1 and B2 belong to the cupin domain-containing proteins or its isoforms. Hence, we analyzed the co-expression status of these genes in *O. sativa* and the Arabidopsis homolog using STRINGdb^34^. The expression pattern revealed a higher co-expression among the B2 proteins, whereas B1 showed a maximum co-expression only with its isoform, Os03g0663800. In Arabidopsis, the co-expression pattern was different as compared with rice. However, in most cases, the target portfolio of the activity-based probe surpasses the number of proteins which can be separated by SDS-PAGE while employing in-gel based ABPP. Besides, it is not possible to visualize low abundance target proteins by this gel-based ABPP method.

### Identification of rice bran serine hydrolases by gel-free ABPP

**Figure 3:**
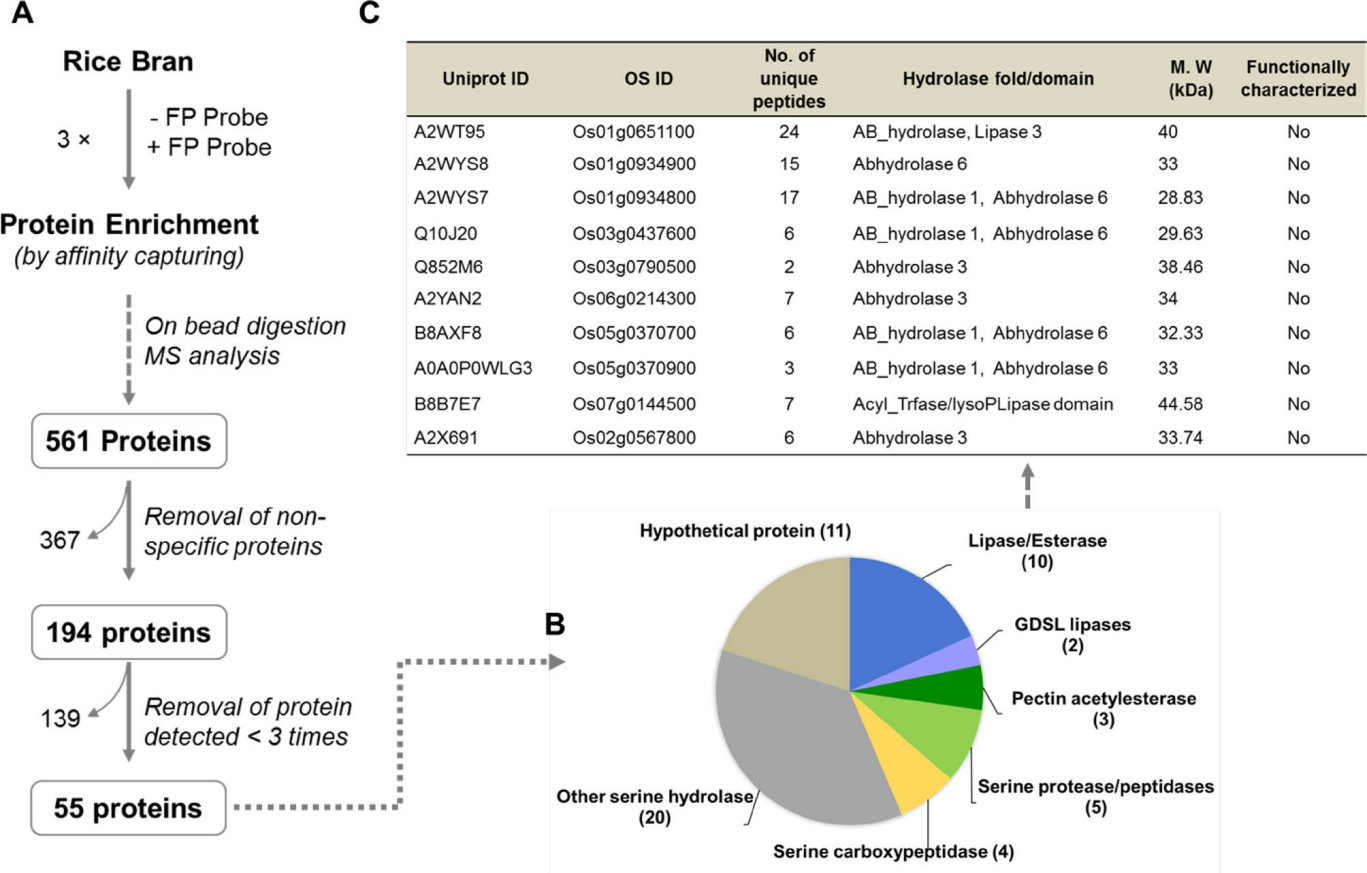
Identification of rice bran serine hydrolases by gel-free ABPP. (**A**) Criteria for selection of serine hydrolase by gel-free ABPP enrichment experiment. Rice bran (RB) protein lysates were pre-labeled with FP-desthiobiotin conjugated probe or DMSO (no probe control). The labeled proteins were enriched by affinity capturing on high-capacity streptavidin agarose resin followed by on-bead trypsin digestion. The possible true serine hydrolases (SHs) were shortlisted by the removal of background proteins (DMSO control) as well as by their detection in at least three times in FP-sample. (**B**) Functional classification of the identified 55 SHs. The identified protein portfolio represents different classes (lipases/esterase, GDSL lipases, pectin acetylesterases, serine protease/peptidase, serine carboxypeptidases, and other serine hydrolases) of SH superfamily including many unannotated proteins. (**C**) List of identified RB lipases/esterases along with their Uniprot and unique OS ID. The number of unique peptides, presence of hydrolase fold/domain, predicted molecular weight of the identified lipases are highlighted.

The inherent limitations of the in-gel fluorescence scanning and gel-based ABPP had led us to perform more sensitive “gel-free formats” to get detailed information about the rice bran SHs. This is the most advanced format of ABPP for the identification of a specific class of functional protein targets^18^. The advantage of gel-free ABPP coupled with on-bead digestion is unparalleled as it provides much higher sequence coverage of the identified proteins, which enhances the confidence level of protein identification. A typical gel-free ABPP, coupled with on-bead digestion, as well as the SHs selection criteria, are shown in Fig. 3A. Here, we used desthiobiotin-FP (biotin-conjugated; FP-Bio; Fig. 1B-ii) for the labeling of RB-SHs followed by enrichment of the labeled proteins on the streptavidin matrix. DMSO was used as no probe control. Through this platform, we were able to map ~ 561 possible active SHs that included the background proteins or the most abundant non-serine hydrolase proteins. There are many proteins that were found repeatedly in both probe and no probe (DMSO treated) samples. These proteins were classified into two groups: Biotin containing endogenous proteins and proteins, which are highly abundant in the sample (discussed below). Among the no probe-target protein, pyruvate decarboxylases (XP_015631876.1), methylcrotonyl-CoA carboxylase (XP_015620588.1), malonyl-CoA carboxylase (XP_015651152.1) which are endogenously biotinylated protein and co-purified along with the target peptides on streptavidin matrix. Finally, after subtracting the background proteins and the low confidence level (detected < 3 times) proteins, we observed a total of 55 SHs (Fig. 3A).The portfolio of the identified proteins was classified into ten lipases/esterase, two GDSL lipases, three pectin acetylesterases, five serine protease/peptidase, four serine carboxypeptidases, twenty other serine hydrolases and ten hypothetical proteins (Fig. 3B). The list of identified lipases/esterases, along with their identity, is shown in Fig. 3C. The conserved domain analysis of these enzymes revealed that they all carry α/β-hydrolase fold that is essential for the hydrolase activity (Fig. 3C).

### Analysis of identified SHs present in the rice bran

**Figure 4:**
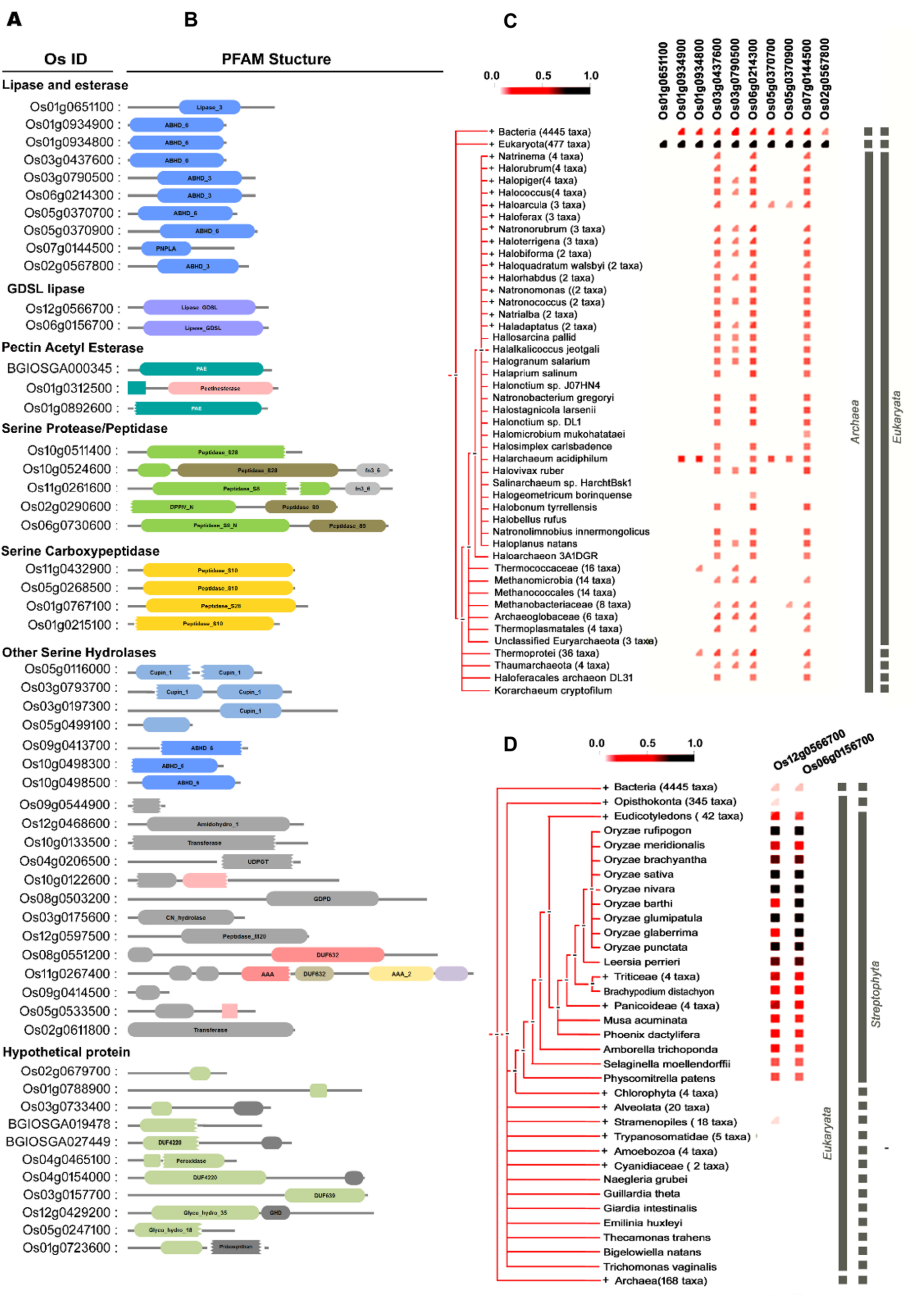
Analysis of identified rice bran serine hydrolases. (**A**) A complete set of the identified serine hydrolases and its classification with their unique OS ID. The profile shows different subclasses of well-known serine hydrolases. (**B**) Predicted PFAM structures of the identified serine hydrolases. (**C**) Clustering, and gene co-occurrence analysis of the identified ten lipase/esterase. (**D**) Clustering, and gene co-occurrence analysis of GDSL lipases. Analysis of gene co-occurrence was performed by constructing a phylogenetic tree using the ‘‘STRINGdb. The phylogeny shows the occurrence of the proteins across species. The genes encoding the proteins are listed on the top of the phylogenetic tree. The intensity of red color represents a degree of conservation of the homologous protein in the species.

**Figure 5:**
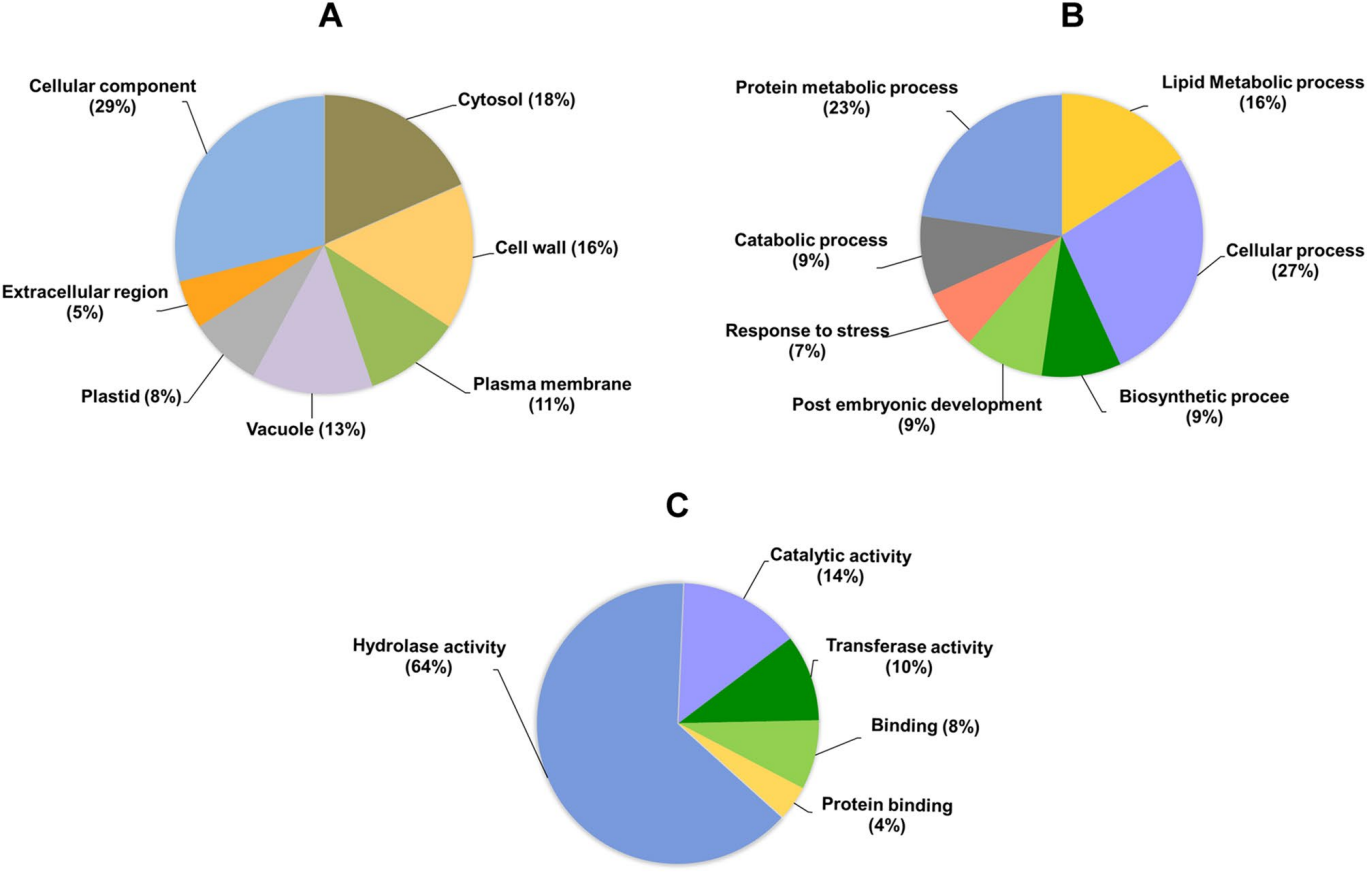
Gene ontology representation of the identified serine hydrolases. Gene Ontology (GO) enrichment analysis of ABPP identified proteins were carried out using AmiGO 2 database and classified under the Gene Ontology categories. The pie charts represent the cellular distribution (**A**), biological process (**B**), and molecular function (**C**) of 55 protein identified as the serine hydrolase. The lipid metabolic (GO: 0006629) and protein metabolic (GO: 0019538) processes are the major biological process. The hydrolase activity (GO: 0016787) is the major molecular function of the identified SHs.

In *O. sativa*, only a small percentage of the proteins encoded by the genome are functionally characterized, and a larger percentage of proteins remain uncharacterized. To get an insight into their functions, we have performed protein family (Pfam) analysis for the 55 SHs proteins unraveled by ABPP along with their predicted domains (Fig. 4A,B). Fifty percentages of the protein fall into well-established SH families such as lipase/esterase, GDSL lipase, protease, serine protease/peptidase, a serine carboxypeptidase, and pectin acetyl esterase. However, there are many proteins categorized as other serine hydrolase and hypothetical proteins. For example, proteins with Domain of Unknown Function (DUF 639, DUF4220), which is functionally active during our ABPP labeling. Protein family analysis depicted that many proteins feature more than one domain. For example, Os10g0524600 has a fibronectin 3 type domain along with the peptidase S8 domain that may play an assisting role for the protein^22^. Further studies are required for the functional annotation of these identified hypothetical proteins. In a quest to understand the biological functions of the identified SHs, we carried out the Gene Ontology (GO) enrichment analysis. The GO enrichment analysis is layered into three different categories, such as molecular function, biological process, and cellular component (Fig. 5A–C). The cellular component annotation of these identified SHs displayed their ubiquitous distribution (Fig. 5A). GO annotation of biological processes confirms their involvement in various metabolic processes. As shown in Fig. 5B, a major portion of the protein is predicted to be implicated in the lipid metabolic process, which is understandable from the fact that there are many active lipases in rice bran. The majority of the identified proteins categorized in GO “molecular function” falls under hydrolase activity, catalytic activity, and transferase activities (Fig. 5C). These SHs belong to different protein families and are representative of diverse biochemical pathways. However, the biochemical functions of the detected serine hydrolases are majorly unknown. In addition, we also analyzed the co-occurrence of the identified lipase/esterases, including GDSL lipase across the genomes of the three kingdoms of life, such as Eukaryota, Archae, and Bacteria using STRINGdb^34^. The phylogenetic distribution of the identified RB SHs showed a ubiquitous occurrence in other genomes of highly divergent species. As shown in the phylogeny (Fig. 4C), all the lipases/esterases are predominantly present across the 447 taxa of eukaryotes, and nine among them were distributed across the 4,445 taxa of bacteria, and three (Os03g0437600, Os06g0214300, Os07g0144500) were evenly distributed among different Archaea. The two GDSL lipases are also distributed among all the species of *O. sativa* (Fig. 4D). These results showed the robustness of the ABPP approach in identifying the highly divergent and widely distributed protein.

### Cupin domain-containing storage protein Os12Ssp encodes protease activity

In addition to the well-known group of SHs, we also identified 31 uncharacterized proteins (56%) that belong to the group in a protein of unknown function. The divergent sequences of these proteins precluded their annotation by their sequence similarity to the well-known template proteins. Hence, we report additional evidence of SH activity for one of the proteins identified by ABPP approach. We have demonstrated the protease activity for Os05g0116000, which is presently annotated as 12S storage protein (designated as Os12Ssp). In general, seed storage proteins (SSP) are considered as metabolically inactive and serve merely as energy reserves for embryonic growth during germination. SSP are amassed in the penultimate stage of seed maturation and are degraded during seed germination for supplying amino acids to developing seedling as a nutritional source^35^. These storage proteins often contain cupin domain, a conserved β-barrel fold (‘*cupa*’ means small barrel in Latin), which was originally found within germin and germin-like proteins in higher plants^36^. The superfamily comprises of 20 families whose members perform various catalytic functions like dioxygenases, hydrolases, decarboxylases epimerases and isomerases^37, 38^. The proteolytic enzymes that hydrolyze the storage proteins are mostly cysteine proteases. However, there are reports which include serine proteases being involved and responsible for the initial degradation of storage protein^39^. Recently the catalytic functions of cupin domain-containing proteins were reported in plants and are involved in various hydrolytic activity, including protease activity^7, 40^. Report also evidence that ABPP is a novel strategy to check seed viability and subsequent germination by monitoring the seed proteome, specifically protease activity^41^.

**Figure 6:**
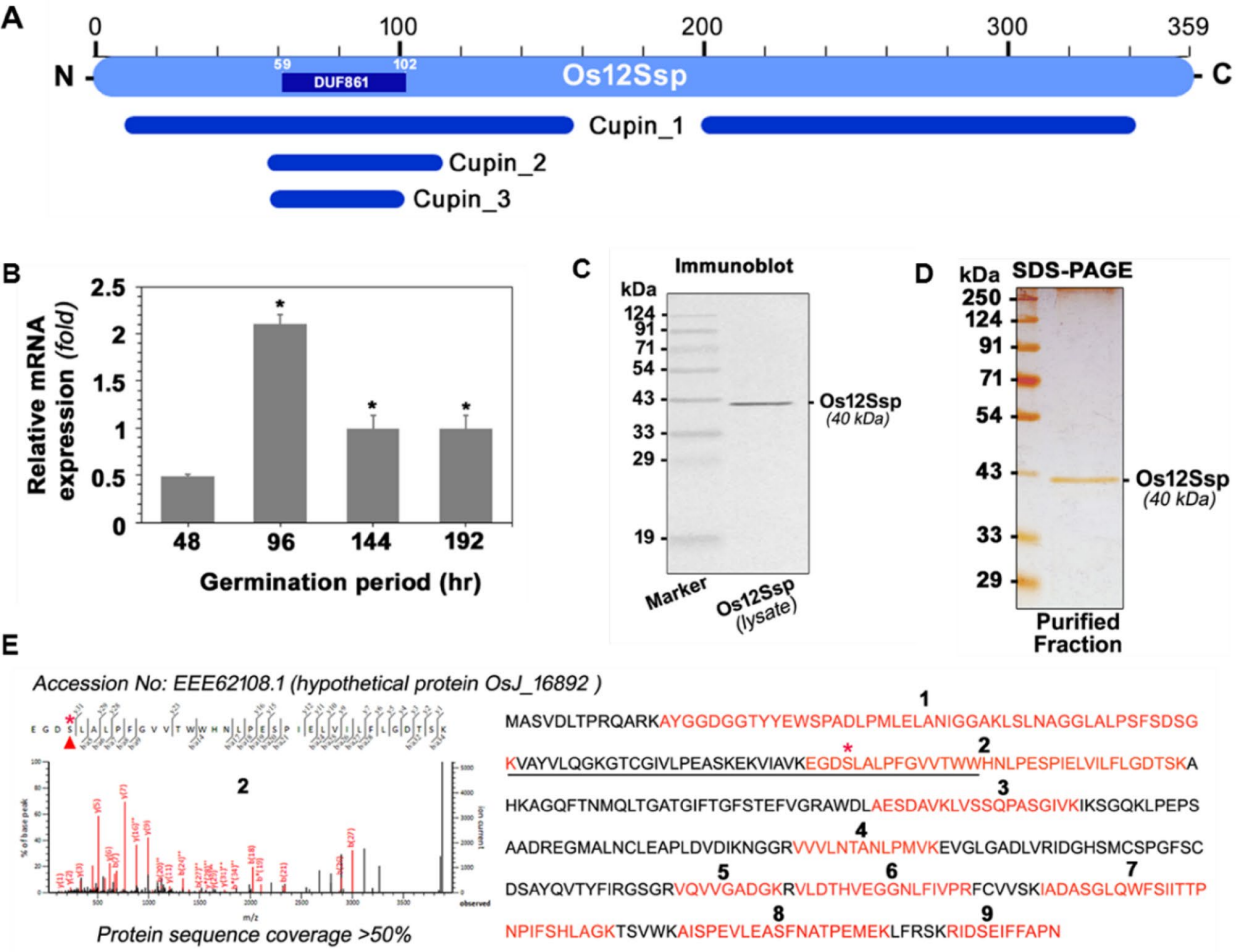
Expression of Os12Ssp protein. (**A**) Schematic representation of domains and motifs of the identified Os12Ssp protein. The presence of different cupin domains was indicated separately. GDSL motif is located within the domain (DUF861) of unknown function (PF05899, a protein of unknown function) at N term. (**B**) Relative mRNA expression of Os12Ssp gene during germination. Actin was used as an internal control. Values are means ± SD of three independent experiments (*p < 0.05). For the biochemical characterization, the coding region of Os12Ssp was amplified from rice cDNA and cloned into the PYES2/NTC vector. The positive clone and its corresponding vector were transformed into *yju3Δ *strain. Protein expression was induced at 0.4 OD (*A*_600_) with the induction medium (SM-U supplemented with 2% galactose and 1% raffinose, w/v) for 24 h followed by recombinant protein purification using Ni^2+^-NTA column chromatography. (**C**) Confirmation of the recombinant expression of Os12Ssp by immunoblot analysis. (**D**) SDS-PAGE analysis of the purified recombinant Os12Ssp. The separated protein was visualized by silver stained. (**E**) Confirmation of the expressed protein by in-gel trypsin digestion. MS/MS fragmentation pattern of the unique peptide is highlighted in red color. *Represents the active site serine residue. A predicted amino acid sequence of the unknown function is underlined.

**Figure 7:**
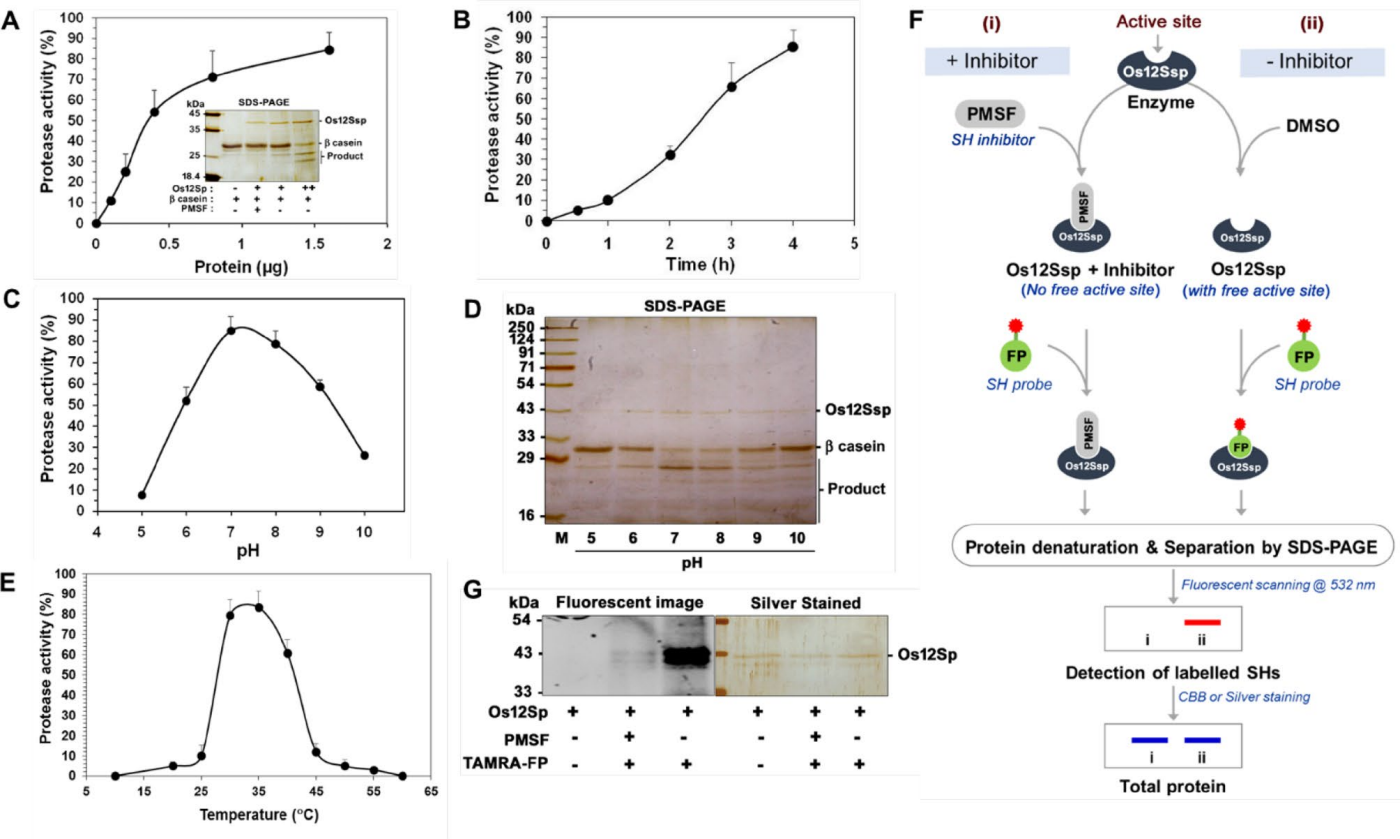
Biochemical characterization of Os12Ssp. The serine protease activity was monitored by the in vitro enzyme under different assay conditions using the purified Os12Ssp protein. The activity was determined in the presence of β-casein (10 μg) as a generic serine protease substrate with purified Os12Ssp as an enzyme source. The hydrolytic product was monitored by separation on a 12% SDS-PAGE followed by silver staining. (**A**) The assay was performed with increasing concentration of protein (0–1.6 µg) 4 h at 37 °C. Inset represents the inhibition of serine protease activity by PMSF (100 µM), a serine protease inhibitor. (**B**) The time-dependent in vitro serine protease activity of Os12Ssp. The reaction was initiated by the addition of 1 µg of protein. (**C**) Assay was performed at different pH with the recombinant Os12Ssp at 37 °C for 4 h. (**D**) A representative silver-stained gel shows the serine protease activity of Os12Ssp. (**E**) The temperature dependence of serine protease activity. (**F**) A schematic representation of Os12Ssp protein active site labeling by competitive ABBP assay. For this, Os12Ssp was pre-incubated with either PMSF (i) or DMSO (ii), followed by active-site labeling with a serine hydrolase probe. Proteins were separated by SDS-PAGE, followed by in-gel fluorescent scanning to detect the labeled Os12Sp. (**G**) Active site labeling of Os12Ssp. The fluorescent signal represents the functional state of the enzymes, and the activity was inhibited by PMSF. Values are means ± SD of three independent experiments.

First, we performed the bioinformatics analysis for possible domains and motif in the protein sequence of Os12Ssp. It was revealed that it contains four domains, which include two cupin 1 domain (Fig. 6A). The cupin domain 2 and 3 were part of N terminal cupin1 domains. The serine hydrolase motif (GDSL like) was located within the DUF861 domain. Further, we analyzed the expression status of Os12Ssp during rice seed germination, since SHs are highly active during germination and facilitate the energy mobilization from stored lipids and proteins^29^. The level of mRNA of Os12Ssp was maximum at 96 h with twofold higher when compared with other time points (Fig. 6B). It is very likely that Os12Ssp may have a significant role during the initial stage of germination, which led us to study the enzyme activity of this protein. We have cloned the full-length CDS of Os12Ssp and heterologously expressed in *Saccharomyces cerevisiae* (Fig. 6C)*.* To confirm the identity of the recombinant protein, the purified protein (Fig. 6D) was subjected to in-gel trypsin digestion followed by Mascot analysis. The obtained unique peptides (n = 9) were matched with native Os12Ssp (Fig. 6E). Biochemical characterization was carried out with recombinant Os12Ssp by in vitro enzyme assays and competitive ABPP. The in vitro serine protease assay was performed at various assay conditions using β-casein as a substrate. The protein-dependent (Fig. 7A) and time-dependent (Fig. 7B) protease activity were observed in the presence of Os12Ssp when compared with no-enzyme controls. The protease activity was inhibited by PMSF, a proven serine protease inhibitor (Fig. 6A, inset). The effect of pH and temperature on the protease activity was monitored at various pH (5 to 10) and temperatures ranging from 10 to 60 °C. Os12Ssp showed maximum activity at pH between 7.0 and 8.0 (Fig. 7C,D) and an optimum temperature of 35 °C although it is active from 30 to 45 °C (Fig. 7E). Further, the serine protease activity was validated by ABPP assay with Os12Ssp in the presence of PMSF (Fig. 7F,G). A typical competitive ABPP workflow is shown in Fig. 7F. The active site labeling was carried out with 1 µg of purified Os12Ssp pre-incubated with PMSF or DMSO, followed by TAMRA-FP. The fluorescent signal was reduced ~ 90% in PMFS treated lane as compared to DMSO. It revealed that Os12Ssp encodes serine protease, and the activity is inhibited by PMSF. The observed result was similar to the enzyme assay. Collectively, our results confirmed that Os12Ssp is a serine protease that was annotated as a storage protein.

## Discussion

Among the cereals, wheat and rice are the most important crops, accounting for over 50% of the global cereal production. Apart from nutritional importance, the rice contains a range of nutraceutical components such as steryl ferulates, phytosterols, tocopherols, and tocotrienols in bran with proven health-promoting activity^2, 3^. Further, the oil extracted from rice bran has a balanced fatty acid composition. The nutritional and nutraceutical components of the crop is highly dependent on the metabolic regulation governed by modulation of various enzyme activities. Among the enzyme classes, serine hydrolases (SHs) play a significant role in several physiological processes at a molecular level. In the present study, we have applied the ABBP approach in an attempt to profile the active SHs present in rice bran. First, the detection and identification of rice bran SHs were performed by gel-based ABPP (In-gel fluorescence scanning), which is the most basic format for the visualization and characterization of probe-labeled proteomes in ABPP experiments^42^. For the ABPP assay, we have removed the lipid fractions from the bran sample to minimize the competition from the endogenous lipids with the serine hydrolase probe. Since lipases belong to the serine hydrolase family, the lipids will eventually weaken the interaction of the probe with the lipase active site and lead to loss of potential targets^43^. The labeling of active serine hydrolases was done using 2 µM FP-Rh at pH 8.0 because the optimal SHs labeling was observed at this pH 8.0 in the complex protein lysates^44^. The organophosphate or -phosphonate compounds as well as carbamate compounds, are the potent SHs inhibitors and have been experimentally proven in competitive ABPP assay in the animal, plant, and archaea model systems^45–47^. To get more insight into the specificity and selectivity of rice bran SHs, we performed in vitro competitive ABBP with known serine hydrolase inhibitors such as PMSF, paraoxon, and profenofos. PMSF is a proven and potent irreversible serine protease inhibitor^48^. In the gel-based ABPP, the two major signals which are inhibited by pre-incubation with PMSF in in-gel fluorescence scanning are, therefore, serine proteases. In our competitive ABPP assay, it was also observed that many of the detected SHs are sensitive towards commercial agrochemicals, paraoxon, and profenofos, which are the potent esterase and acetylcholinesterase inhibitors respectively^45, 47^. The gel-based competitive ABPP results revealed the presence of many active esterases/lipases in the RB proteome. Since the in-gel fluorescence ABPP does not assign any identity to the probe-labeled target proteins, enrichment experiments were followed using an anti-probe antibody^33^ in the immuno pulldown experiment. The in-gel trypsin digestion of enriched proteins provides a direct link between the labeling patterns of in-gel fluorescence signals and their identities^49^. Through this approach, two major proteins were identified as cupin domain-containing proteins (Fig. 2A). The B1 protein was identified unequivocally and by the gene ID Os03g0793700. In the earlier study, it was reported that this gene is highly expressed during germination along with the cupin protein family^50^. Recently, catalytic protease function was demonstrated for a cupin domain-containing protein in rice bran^7^. Collectively, from our gel-based ABPP result, we infer the B1 to be a catalytic protease with a cupin domain and may have a physiological role during the germination of rice. The B2 protein that was also identified as a cupin protein is multimeric in nature. It is not surprising considering the fact that cupin proteins contain multiple domain^37^. Even though an enzyme is annotated as other than an SH, they still function as an SH because its serine hydrolase activity may be embedded in another domain^51^. The co-expression analysis of B1 and B2 proteins in rice and Arabidopsis show a distinct pattern (Fig. 2B). In Arabidopsis, the co-expression pattern was different as compared with rice. It was reported that divergent expression patterns are due to the differential sequence evolution and duplication of genes, which tend to acquire more distinct functions, particularly in dicot-monocot species^22^. The gel-free format of ABPP facilitates the analysis of probe labeled peptides from a digested sample rather than intact proteins, which in turn allow protein size-independent separation of targets, ruling out a major limitation of the gel-based ABPP^33^. Besides, the affinity tagged active-site peptides still bound to the beads can optionally be eluted and further analyzed by mass spectrometry for active site characterization^51^. However, in such cases, stringent selection criteria have to be adopted along with sufficient replication and controls to rule out any background proteins. In our approach, we have also encountered many nonspecific proteins as putative targets. The major abundant proteins, to name a few, are enzymes of glycolysis and TCA cycles as well as other abundant proteins such as aldose reductase (XP_015639340.1) present in mature rice seeds, alcohol dehydrogenase (ADH03849.1) present in rice endosperm and embryo^52, 53^. In *O. sativa*, only a small percentage of the proteins encoded in the genome is functionally characterized, and a larger percentage of proteins remain uncharacterized. In such a case, clustering is used as a first step to minimize the complexity of biological networks by classifying them into specific functional family^54^. In the present study, fifty percentages of the protein, identified by ABPP, fall into well-established SH families. However, there are many proteins categorized as other serine hydrolase and hypothetical proteins based on their structural similarity with well-known template proteins (Fig. 4A,B). In many cases where probe binding and sequence-based annotations disagree, the proteins might indeed be SHs that are currently misannotated^51^. For example, Os12Ssp annotated as a storage protein, which was identified in this study by ABPP, and we have provided experimental evidence for its serine protease activity. In general, seed proteins are classified as storage, structural, and metabolic proteins, which are interspersed with more than one of these classes^55^. Seed storage proteins are further classified as prolamins, 2S albumins, 7–8S globulins, and 11–12S (S, sedimentation coefficient) globulin. The cupin family proteins perform diverse functions ranging from enzymatic activities to non-enzymatic features (binding to auxin, transcriptional factors, and seed storage)^37, 56^. For immediate initiation of storage protein hydrolysis by active proteases during reserve mobilization during germination, plants deposits active proteases in the very same compartments where storage proteins are sequestered^28^. The most critical features of an enzyme lie in its stability as well as catalytic performance^57^. The catalytic efficiency of the enzymes highly depends on many factors, including physical (temperature and pH) or chemical (the presence of inhibitors or activators)^58^. In our quest to understand the optimum activity of Os12Ssp, we conducted the enzyme activity under various conditions, and the maximum activity was observed at pH 7, and an optimum temperature of 35 °C. In a similar fashion, Khan et al.^59^ reported a seed-specific serine protease from *Holarrhena antidysenterica,* which was active in neutral to alkaline pH and optimally active at 35 °C towards β casein. Next, we have sought to understand the sensitivity of the purified Os12Ssp towards PMSF. Since ABPP is an active site targeted serine hydrolase (in the present study) identification tool, the identified Os12Ssp must have sensitivity towards PMSF. Similarly, we have observed a strong inhibition of fluorescence signal by PMSF in in-gel fluorescence competitive ABPP assay and abolishes the signal by ~ 90%, which is evident in Fig. 7G. Based on our functional ABPP experiment along with the in vitro protease assay with recombinant protein, we have provided strong evidence that Os12Ssp is a serine protease that was annotated as a storage protein. To the best of our knowledge, this is the first report of cloning and biochemical characterization of a catalytic/hydrolytic 12S seed storage protein from rice.

**Figure 8:**
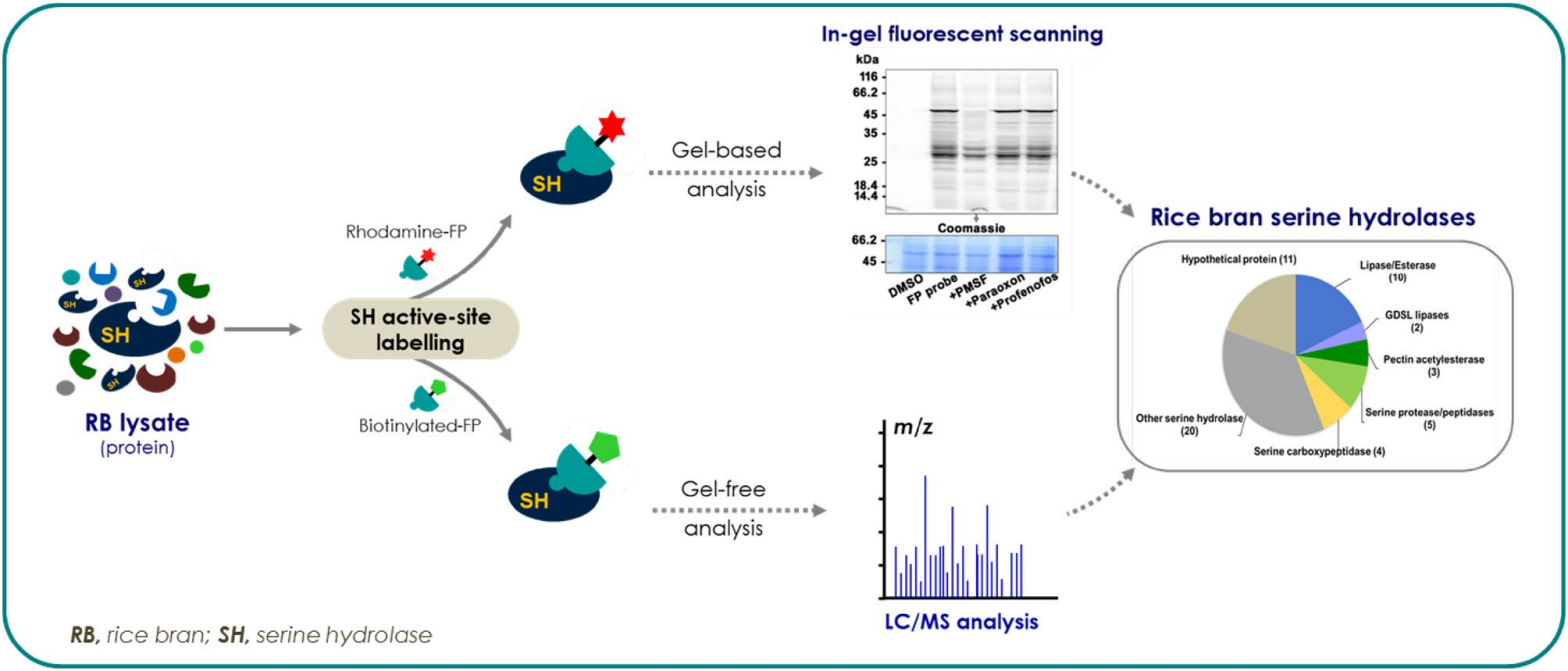
Schematic representation of activity-based protein profiling of rice bran serine hydrolases.

In summary, in the present study, we identified 55 serine hydrolases by gel-based, and gel-free ABPP approaches. The identified SHs are classified under various categories based on the domain and motif organizations. The identified SHs are distributed in different cellular localization, involve in the distinct metabolic process, and perform various molecular functions. The schematic representation of the present study was shown in Fig. 8. Further, the biochemical characterization of an identified Os12Ssp was carried out with the recombinant protein. Collectively, the compressive knowledge generated from this study would be useful in expanding the current understanding of rice SHs and its role in plant growth and development as well as for crop improvements.

## Methods

### Chemicals and reagents

ABPP ActivX Serine Hydrolase Probes ActivX TAMRA-FP (cat no. 88318) and ActivX Desthiobiotin-FP (cat no. 88317), Zeba spin desalting columns, 5 ml (cat no. 89892), high capacity streptavidin agarose (cat no. 20349), anti-TAMRA monoclonal antibody (cat no. MA1-041), cDNA Synthesis Kit, restriction endonucleases and T4 DNA ligase enzyme were procured from Thermo Scientific (USA). Dimethyl Sulfoxide (Cat no. D2650), Dithiothreitol (cat no. D9779), Iodoacetamide (cat no. I6125), MS grade trypsin from porcine pancreas (cat no. T6567), Trifluoroacetic acid (cat no. T6508) were purchased from Sigma Aldrich (USA). LCMS grade acetonitrile (cat no. 9829-03) and water were procured from JT baker Avantor. Urea (cat no.194857) was ordered from MP Biomedicals, Mumbai, India.

### Protein extraction from rice bran

Rice bran was separated from freshly harvested rice (*Oryza sativa*, IR64) seeds and immediately processed for protein extraction. The bran protein extraction was performed by mixing the bran with extraction buffer [(1:10 w/v) consist of 50 mM Tris–HCl (pH 8.0), 150 mM NaCl, 1 mM MgCl_2_, 1 mM KCl, 10% glycerol] and by vortexing the mixture for 30 min at 4 °C. The crude cell-free lysate was obtained by centrifugation at 3,000×*g* for 10 min at 4 °C. Further, the soluble and membrane fractions were obtained by ultracentrifugation at 100,000×*g* for 90 min at 4 °C. After the centrifugation, the top lipid layer was discarded. The membrane fraction was washed twice with the extraction buffer and solubilized with an extraction buffer. Protein estimation was carried out by the Bradford method^60^.

### Activity-based protein labeling and detection of rice bran serine hydrolases

Labeling of active serine hydrolases was performed in rice bran proteins using ActiveX-TAMRA FP, rhodamine-conjugated fluorophosphonate probe (FP-Rh). The assay mixture contains a 2 μM FP-Rhserine hydrolase probe with 100 µg of protein in the total volume of 50 µl of the extraction buffer. The reaction mixture was incubated for 60 min at 37 °C and terminated by the addition of 10 μl of 4 × loading buffer (200 mM Tris–HCl, pH 6.8, 400 mM DTT, 8% SDS, 0.04% bromophenol blue, and 40% glycerol) and boiled for 5 min. Proteins were resolved on a 12% (w/v) SDS-PAGE, and the probe labeled enzymes were detected under fluorescent scanning at 532 nm (Typhoon FLA9500 phosphor imager, GE Healthcare Life Sciences). Further, the gels were stained with Coomassie Brilliant Blue R-250 (CBB), and the images were documented. The stock serine hydrolase probe was prepared in DMSO.

### Competitive ABPP study

To determine the sensitivity of the detected serine hydrolases towards well-known inhibitors of SHs, we performed the competitive ABPP labeling with known serine hydrolase inhibitors such as PMSF, Paraoxon, and Profenofos. Competitive serine hydrolase labeling was conducted with 100 µg of soluble protein by pre-incubation with either 100 μM inhibitor or DMSO (as a control) for 30 min at 37 °C. After incubation, 2 μM of the probe was added into each reaction mixture and kept for 1 h at 37 °C in the dark. Separation and detection of ABPP labeled proteins were done as described above.

### Immunoaffinity enrichment of rice bran serine hydrolases

Rice bran serine hydrolases were enriched by an immune-pulldown assay using an anti-FP probe antibody. The rice bran protein (100 μg) was labeled with 5 μM ActiveX-TAMRA FP or DMSO (as no probe control) for 1 h at 37 °C in the total volume of 250 µl in the dark. After labeling, 5 μl of anti-TAMRA monoclonal antibody was added and kept it for 6 h at 4 °C in the tube rotator (10 rpm). The enzyme-antibody complex was captured by adding 100 μl of protein A agarose and incubated overnight at 4 °C, followed by centrifugation at 1,000*g* for 2 min to collect the beads. The beads were washed extensively with equilibration buffer containing Tween-20 (0.1%) to remove the unconjugated protein. The labeled proteins were eluted and resolved onto a 12% SDS-PAGE, followed by CBB staining.

### In-gel trypsin digestion

Protein bands of interest were excised from the CBB-stained gels (named as B1 and B2), and the destained gel was subjected to in-gel trypsin digestion. In short, gel pieces were washed three times with 500 μl of wash solution (50% acetonitrile, 50 mM ammonium bicarbonate) for 1 h at room temperature in the tube rotator. The gel was dehydrated with 100% acetonitrile for 5 min, dried under vacuum concentrator followed by rehydration with 150 μl of reduction solution (10 mM DTT, 100 mM ammonium bicarbonate) for 30 min at 65 °C. Rehydrated gel pieces were alkylated with 100 μl alkylation solution (100 mM ammonium bicarbonate, 50 mM iodoacetamide) for 30 min at room temperature in the dark. Gel pieces were washed three times with 500 μl of wash solution and dried under a vacuum concentrator. Trypsin digestion was performed with 0.5 ng of MS grade trypsin in 100 μl of 50 mM ammonium bicarbonate solution for 16 h at 37 °C with constant shaking. The tryptic fragments were collected by centrifugation at 1,300*g* for 2 min, and the supernatant was transferred into a sterile centrifuge tube. Further, residual peptide fragments were extracted from gel pieces with 25 to 50 μl of extraction solution (60% acetonitrile, 0.2% TFA) by vortexing followed by centrifugation. Finally, all the extracts were pooled together and dried under a vacuum concentrator.

### Gel free ABPP coupled with on-bead trypsin digestion of enriched proteins

The biotin-conjugated serine hydrolases probe was used for labeling and enrichment/identification of rice bran proteome^61^. In brief, one mg (500 µl) of protein extracts were labeled with 20 µM of desthiobiotin conjugated FP-Probe by incubating them at 37 °C for one hour. The reaction was stopped by adding 500 μl of 10 M urea followed by a reduction of the sample with 500 mM DTT (incubation at 65 °C for 30 min) and alkylation with 1 M iodoacetamide in the dark. The proteins were desalted using a desalting spin column. The labeled proteins were enriched by affinity capturing by incubating with 70 μl of 50% high capacity streptavidin agarose for 1 h in tube rotator. Next, the beads containing the labeled proteins were separated by centrifugation at 1,000×*g* for 1 min. The unbound proteins were removed by washing with 1.5 ml of washing buffer (25 mM Tris, pH 8.0., 150 mM NaCl, 1% NP 40, 1 mM EDTA, 5% glycerol), PBS and LC–MS grade water. The bound proteins were then digested with 10 μg of MS grade trypsin in 250 μl of digestion buffer (2 M urea, 20 mM Tris, pH 8.0) for 16 h at 37 °C with constant shaking. The digested peptides were collected by centrifugation at 1,000*g* for 1 min, and the supernatant was dried in a vacuum concentrator (Eppendorf). Digested peptides were resuspended in a buffer that contains 50% acetonitrile and 0.1% TFA. The peptides were then desalted using C_18_ resin pre-packed micro tips (ZipTip, Millipore). Prior to peptide loading, ZipTip was equilibrated with 100% acetonitrile, followed by 0.1% formic acid. The desalting of peptides was performed by pipetting the sample up and down and six times washed with LCMS water, followed by 0.1% formic acid. The peptides were then eluted by pipetting up and down in the elution buffer (60% acetonitrile/0.1% formic acid). The purified eluted samples were further lyophilized and dissolved in 10 µl of 2% acetonitrile/0.1% formic acid. Finally, the trypsin digested peptides were identified by mass spectrometry.

### LC–MS/MS analysis and identification of enriched peptides

The LC–MS/MS analysis of peptide was carried out in Thermo LTQ Orbitrap, which was coupled with an EASY nano LC1200 system (Agilent). The pre-column used for the liquid chromatography (LC) analysis is Pep map TM 100; 75 µm × 2 cm; Nanoviper C18, 3 µm; 100 Å. The nanoviper fitting is capable of withstanding pressure up to 1,000 bar and provides virtually zero dead volume. The LC analytical column used for this study was an EASY SPRAY PREAMP (RSLC C18 3 µm; 50 cm × 75 µm; 100 Å) silica column, which was tailored for high-resolution separation of peptides. The column temperature was set to 35 °C. The LC solvent system consisted of two mobile phases: Solvent A (0.1% Formic acid in HPLC grade water) and Solvent B (80% acetonitrile and 0.1% formic acid in HPLC grade water). The desalted peptides were directly loaded onto the analytical column. Next, the peptides were separated on the analytical column by running a 180 min gradient of solvents A and B (Start with 10% B for 2 min, gradient 10 to 45% B for 166 min, gradient 45 to 95% up to 172 min). The flow rate was limited to 250 nl/min. The mass spectrometer was performed in positive ion mode. Precursor ion scanning was done in an orbitrap analyzer in the scan range of 375–1,700 at a resolution of 120,000 with a maximum ion injection time of 50 ms. Product ion spectra were monitored in an ion type analyzer. The ionization potential voltage was set to 1.8 kV. For fragmentation, high collision energy (CID) was set to 30% for the generation of MS/MS spectra, which facilitate peptide sequencing and identification. For the MS/MS events, an ion trap detector was used. MS^2^ scan range (m/z) was set to 100–2000, with a maximum ion injection time of 35 ms.

### Peptide and protein identification using Mascot

The MS^2^ spectra data were extracted and processed for protein identification. The raw data files were exported as Mascot Generic File (MGF). For peptide and protein identification, the MS/MS spectra data were searched using a Mascot search engine (version 2.7.10) against the *Oryza sativa* taxonomy filter. For the Mascot search, the following parameters were included: Fixed modification of carbamidomethyl (57.02146 Da); variable modifications of acetyl (protein N-term) (42.01057 Da); oxidation of methionine (15.99492 Da). Enzyme specificity was set to Trypsin/P, and the maximum missed cleavage allowed was 2. The peptide mass tolerance was set to 10 ppm, and fragment mass tolerance was set to 0.6 Da. Ion score cut off was set to 30. The significant threshold chosen was 0.05.

### Gene ontology, Co-occurrence, and Co-expression analysis

Gene Ontology (GO) enrichment analysis of the genes encoding the corresponding protein identified by ABPP was carried out using the “AmiGO 2 database’’. Gene co-expression and gene co-occurrence analysis of the identified genes, as well as the co-occurrence of these genes across genomes, were analyzed using “STRING: functional protein association networks’’. Gene co-occurrence analysis showed the functional association among proteins. This phylogenetic profile was based on the premise that functionally linked proteins generally co-occur in genomes.

### Cloning, expression, and purification of Os12Ssp

A full-length coding sequence of the Os12Sspgene was amplified from cDNA using gene-specific primers. For recombinant protein expression, the ORF was cloned into the pYES2/NTC vector using *BamHI* and *EcoRI* restriction sites. Positive clone harboring the gene was confirmed by both restriction digestion and by nucleotide sequencing. The Os12Ssp gene construct and empty vector were transformed into yeast lipase mutant strains, yju3Δ using the lithium acetate, and transformants were selected by uracil depletion media (SM-U)^62^. The construct was precultured at 30 °C in 5 ml of SM-U + 2% (w/v) dextrose medium for overnight, and the cells were collected by centrifugation and washed with sterile water. Protein expression was induced at 0.4 OD (*A*_600_) in the induction medium (SM-U supplemented with 2% (w/v) galactose and 1% (w/v) raffinose for 24 h. Cells were harvested at 3,000×*g* at 4 °C for 5 min. Pellets were washed with sterile water and resuspended in lysis buffer (50 mM Tris–HCl, pH 8.0, and 300 mM NaCl, 2 mM MgCl_2_, 10% glycerol (v/v), and 1 mm PMSF). The cell lysate was centrifuged at 3,000×*g* at 4 °C for 5 min, and the cell-free extract was further centrifuged at 1,300*g* at 4 °C for 30 min. The membrane fraction was solubilized with n-dodecyl-β-D-maltopyranoside (DDM), and the recombinant protein was purified using Ni^2+^-NTA affinity chromatography followed by protein dialysis in phosphate buffer. Os12Ssp expression was confirmed by immunoblot analysis using an anti-His^6^ tag monoclonal antibody.

#### In vitro protease assay

The serine protease activity was measured by monitoring the hydrolysis of β-casein^51^ using recombinant Os12Ssp. The assay mixture consisted of 50 mm Tris–HCl (pH 7), 10 μg β-casein, and the enzyme source (0–1.6 μg) in a total volume of 100 μl. The reaction was conducted at 40 °C for different time intervals (0–4 h) and stopped by the addition of 4 × SDS-PAGE loading buffer and heating at 95 °C for 5 min. The pH and temperature-dependent assay were performed for 4 h in the presence of 1 µg protein for various pH and temperature, respectively. For inhibition study, 100 µM PMSF was as a serine protease inhibitor. The product profile was monitored by separating them on a 12% SDS-PAGE, and visualizing by silver staining.

### Competitive ABPP analysis of Os12Ssp

Competitive serine hydrolase labeling was conducted with 1 µg of Os12Ssp by pre-incubation with either 10 μM PMSF or DMSO (as a control) for 30 min at 37 °C. After incubation, 2 μM of the probe was added into each reaction mixture and kept for 1 h at 37 °C in the dark. Separation and detection of ABPP labeled protein were done as described above. After fluorescent scanning, the gel was silver stained.

## Supplementary information

Supplementary Information.
